# Metagenomic Analysis of Plant Viruses Associated With Papaya Ringspot Disease in *Carica papaya* L. in Kenya

**DOI:** 10.3389/fmicb.2020.00205

**Published:** 2020-03-04

**Authors:** Naomi Nzilani Mumo, George Edward Mamati, Elijah Miinda Ateka, Fredah K. Rimberia, George Ochieng’ Asudi, Laura M. Boykin, Eunice M. Machuka, Joyce Njoki Njuguna, Roger Pelle, Francesca Stomeo

**Affiliations:** ^1^Department of Horticulture and Food Security, Jomo Kenyatta University of Agriculture and Technology, Nairobi, Kenya; ^2^Department of Biochemistry, Microbiology and Biotechnology, Kenyatta University, Nairobi, Kenya; ^3^Department of Plant Physiology, Faculty of Bioscience, Matthias-Schleiden-Institute, Friedrich Schiller University Jena, Jena, Germany; ^4^ARC Centre of Excellence in Plant Energy Biology, School of Molecular Sciences, The University of Western Australia, Perth, WA, Australia; ^5^Biosciences Eastern and Central Africa-International Livestock Research Institute Hub, Nairobi, Kenya

**Keywords:** papaya, next-generation sequencing, *Carlavirus*, *Potyvirus*, ringspot disease, diagnostic primers

## Abstract

*Carica papaya* L. is an important fruit crop grown by small- and large-scale farmers in Kenya for local and export markets. However, its production is constrained by papaya ringspot disease (PRSD). The disease is believed to be caused by papaya ringspot virus (PRSV). Previous attempts to detect PRSV in papaya plants showing PRSD symptoms, using enzyme-linked immunosorbent assay (ELISA) and reverse transcriptase-polymerase chain reaction (RT-PCR) procedures with primers specific to PRSV, have not yielded conclusive results. Therefore, the nature of viruses responsible for PRSD was elucidated in papaya leaves collected from 22 counties through Illumina MiSeq next-generation sequencing (NGS) and validated by RT-PCR and Sanger sequencing. Viruses were detected in 38 out of the 48 leaf samples sequenced. Sequence analysis revealed the presence of four viruses: a *Potyvirus* named Moroccan watermelon mosaic virus (MWMV) and three viruses belonging to the genus *Carlavirus.* The *Carlaviruses* include cowpea mild mottle virus (CpMMV) and two putative *Carlaviruses*—closely related but distinct from cucumber vein-clearing virus (CuVCV) with amino acid and nucleotide sequence identities of 75.7–78.1 and 63.6–67.6%, respectively, in the coat protein genes. In reference to typical symptoms observed in the infected plants, the two putative *Carlaviruses* were named papaya mottle-associated virus (PaMV) and papaya mild mottle-associated virus (PaMMV). Surprisingly, and in contrast to previous studies conducted in other parts of world, PRSV was not detected. The majority of the viruses were detected as single viral infections, while a few were found to be infecting alongside another virus (for example, MWMV and PaMV). Furthermore, the NGS and RT-PCR analysis identified MWMV as being strongly associated with ringspot symptoms in infected papaya fruits. This study has provided the first complete genome sequences of these viruses isolated from papaya in Kenya, together with primers for their detection—thus proving to be an important step towards the design of long-term, sustainable disease management strategies.

## Introduction

Papaya (*Carica papaya* L.) is an important fruit crop both in the tropical and subtropical regions (Mishra et al., 2007). The fruit crop is ranked fourth worldwide among tropical fruits, after banana, mango, and pineapple (Edward and Ballen, 2015). Ripe fruits are rich in vitamins A and C, and based on the recommended daily allowance for these vitamins, papaya is ranked first among the 38 most common fruits (Ming et al., 2008). The levels of vitamins A and C from one medium-sized papaya (whose edible portion is about 350 g) exceed the Dietary Reference Intakes of 3000 IU for vitamin A and 90 mg for vitamin C, as established by the United States Food and Nutrition Board for adult minimum daily requirements (Ming et al., 2008). Consumption of this fruit is important in preventing vitamin A deficiency, a cause of childhood blindness in many developing countries, including Kenya (Oyunga et al., 2016). The fruit is reasonably priced and is rich in nutrients, making it a “common man’s” fruit. Papaya is also a source of papain, a proteolytic enzyme obtained by collecting and drying the latex exuded from scratches on the surfaces of slightly immature papaya fruits. The enzyme is purified and used in foods, beverages, pharmaceuticals, and by manufacturing industries (Yogiraj et al., 2014).

In Kenya, the fruit crop is grown in many regions (mostly by small-scale farmers for subsistence), and is ranked sixth for its economic importance after the banana, pineapple, mango, avocado, and watermelon (Asudi, 2010; HCDA, 2016). Large quantities of fruits are mainly consumed locally as a dessert or used in making jams and purée, while the leaves serve as compost. There are few large-scale farmers who produce the fruit as a source of income for local and export markets (Asudi, 2010).

Despite its importance, national economies of many papaya-growing nations are jeopardized by the papaya ringspot disease (PRSD). The disease affects papaya plants at all stages of growth and naturally spreads very quickly, leading to infection of the whole orchard within 3–7 months with severe yield losses of up to 100% (Ventura et al., 2004; Tripathi et al., 2008; Sharma and Tripathi, 2014). A characteristic symptom of the disease on infected plants is the production of ringed spots on the fruits (Gonsalves, 1998; Sharma et al., 2014). Other symptoms of the disease include vein clearing, mottling, mosaic chlorotic spots, leaf curling, green blisters, and distortion of leaves termed as “shoe-stringing.” Reduction in size of the leaf canopies, as the disease advances, results in stunted growth of the plant. Irregular oily, or water-soaked streaks or marks are seen on stems and leaf petioles. These symptoms can occur together or separately. Fruits affected by this disease are of poor quality with low sugar levels, attracting low prices both at local and export market levels (Tripathi et al., 2008; Sharma and Tripathi, 2014). In addition, if plants are infected with the disease—either at the seedling stage or within 2 months after planting—they fail to produce mature fruits, and the affected papaya orchards have a short lifespan of less than a year (Gonsalves, 1998; Tennant et al., 2007). The impact of the disease on rural farming communities has been extreme, due to the fact that papaya trees can no longer be grown without a high possibility of being damaged (Sakuanrungsirikul et al., 2014). The disease is known to be caused by papaya ringspot virus (PRSV), a *Potyvirus* in the family *Potyviridae* (Tripathi et al., 2007, 2008; Sharma and Tripathi, 2014).

A study conducted in Kenya documenting papaya cultivation between 2008 and 2009 reported PRSD as the main constraint to papaya production in several regions of the country, including the Coast, Central, Rift Valley, Western, and Eastern regions (Asudi, 2010; Ombwara et al., 2014). Several attempts to detect PRSV in papaya plants that showed PRSD symptoms using double antibody sandwich enzyme-linked immunosorbent assay (ELISA) were not conclusive (Ombwara et al., 2014). Reverse transcriptase-polymerase chain reaction (RT-PCR) procedures using published PRSV primers (Hema and Prasad, 2004; Jain et al., 2004; Diallo et al., 2008; Omar et al., 2011; Srinivasulu and Sai Gopal, 2011; Mohammed et al., 2012; Martínez et al., 2014) also failed to detect the virus. The same failure occurred using primers designed based on PRSV sequences available in GenBank to amplify the virus in symptomatic plants (unpublished data). This has resulted in the notion that there could be a different strain of PRSV in Kenya, or a different virus(es) infecting papaya in the country.

With the development of next-generation sequencing (NGS) technology, plant virus discovery, diagnostics, and evolutionary studies have increased and improved enormously (Roossinck, 2017). The technology can be used to identify plant viruses in a given sample with or without prior knowledge of the viral types present, and it can also reveal the presence of novel and unsuspected agents. The approach has also been helpful for viral co-infection detection in many plants (Candresse et al., 2014; Roossinck, 2015; Akinyemi et al., 2016; Blawid et al., 2017). Therefore, we used NGS approach coupled with RT-PCR and Sanger sequencing to identify and characterize the virus(es) causing symptoms associated with ringspot disease on papaya in Kenya. We believe this to be the first application of NGS technology in assessing viruses associated with PRSD infecting this important fruit crop in Kenya and that it is likely to help in the design of long-term and sustainable disease management strategies in the country.

## Materials and Methods

### Sample Collection

**FIGURE 1:**
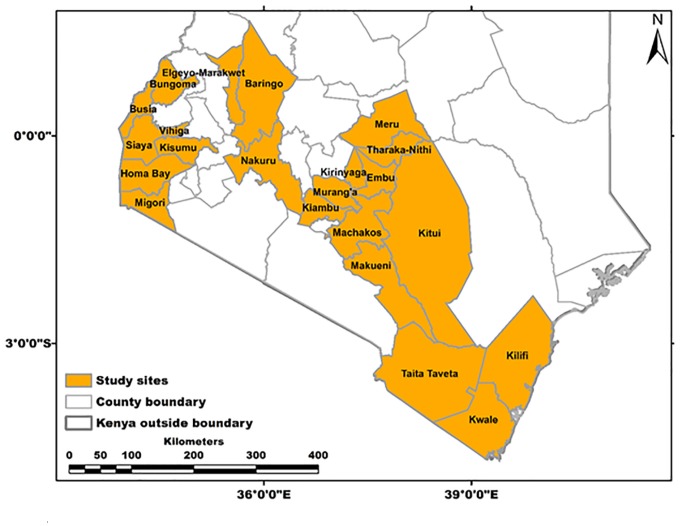
Counties in Kenya sampled during this study.

Field surveys and sampling were carried out during February–April of 2017 in 22 administrative regions (counties) in Kenya—namely, Taita Taveta, Kwale, Kilifi, Bungoma, Busia, Siaya, Vihiga, Kisumu, Homabay, Migori, Nakuru, Baringo, Elgeyo Marakwet, Kiambu, Murang’a, Kirinyaga, Embu, Tharaka Nithi, Meru, Makueni, Machakos, and Kitui (Figure 1). These counties were selected based on reported papaya production and the presence of the PRSD-related symptoms (Asudi, 2010; Ombwara et al., 2014). A total of 287 leaf samples (200 with PRSD symptoms and 87 symptomless) were collected from randomly selected plants using sterile forceps and then immediately immersed in RNA*later*^®^ (Invitrogen^TM^) solution to prevent the degradation of RNA. The samples were then transported to the Biosciences eastern and central Africa–International Livestock Research Institute (BecA-ILRI) Hub in Nairobi, Kenya, and stored at 4°C until RNA extraction. Forty-eight samples (34 with and 14 without ringspot disease symptoms) were randomly selected for NGS analysis based on the region, and symptoms were observed. In every county, a representative sample with or without symptoms was selected, and in counties where more than one sample was chosen, differences in symptoms exhibited by the plants were considered. The geographic coordinates of the sample collection sites were obtained using a handheld global positioning system (GPS), and the data were converted into GIS using ARCGIS 10.4 (Figure 1).

### RNA Extraction, Library Preparation, and Illumina MiSeq Sequencing

Leaf samples selected for NGS analysis were dried using a clean, absorbent paper towel to remove the RNA*later*^®^ and then powdered in liquid nitrogen with sterile mortars and pestles. Total RNA was extracted from 0.1 g of leaf samples using the RNeasy^®^ plant mini Kit (Qiagen Inc.), following the manufacturer’s instructions. The RNA was eluted in 70 μl of RNase-free water, visualized on a 0.8% (w/v) agarose gel electrophoresis at 100 V for 30 min, and its quantity was measured using the ssRNA assay on the Qubit^®^ 2.0 fluorometer (Invitrogen^TM^) system. The extracted RNA was then stored at −80°C. The cDNA libraries were prepared from 1 μg of the total RNA using the Illumina TruSeq^®^ RNA sample preparation protocol according to the manufacturer’s instructions (Illumina, San Diego, CA, United States). Briefly, poly-A containing mRNA molecules were purified using oligo-dT, and then fragmented into small pieces using the Illumina “Elute, Prime, Fragment Mix.” The fragmented RNA was copied into the first-strand cDNA using reverse transcriptase and random primers, and second-strand cDNA was synthesized using DNA polymerase I and RNase H. The double-stranded cDNA was purified using Agencourt AMPure^®^ XP magnetic beads (Beckman Coulter, Inc., Indianapolis, IN, United States). The end-repair of synthesized cDNA was performed using End Repair mix. Thereafter, 3′ ends were adenylated and unique adaptors for each library ligated to the 5′ and 3′ ends ds cDNA. The dsDNA was enriched through PCR to create the final cDNA library under the following cycling conditions: one cycle of 98°C for 30 s; 15 cycles of 98°C for 10 s; 60°C for 30 s; and 72°C for 30 s, with a final extension of 72°C for 5 min.

The final size and concentration of the cDNA libraries were estimated with the Agilent Tape Station 2200 system (Agilent Technologies, Santa Clara, CA, United States) and Qubit^®^ 2.0 fluorometer (Invitrogen^TM^), respectively. The cDNA libraries, each with unique adaptor, were normalized to 4 nm and pooled for multiplex sequencing. A pooled library consisted of 24 biological samples, each at equal molar concentration (hence, two flow cells) were used. The libraries were sequenced using a 2 × 300 cycle PE V3 Illumina kit (Illumina, San Diego, CA, United States). Paired-end reads were generated using the Illumina MiSeq System at the BecA-ILRI Hub in Nairobi, Kenya.

### RNA Sequence Processing and *de novo* Assembly

Paired-end reads generated in the Illumina MiSeq System were checked for quality using FastQC. The low-quality reads and sequencing adapters were removed using Trimmomatic V 0.33 (Bolger et al., 2014). The host genome was removed by mapping all the reads to the papaya plant genome (GenBank accession number ABIM01000000) (Ming et al., 2008), using Bowtie2 V 2.2.8 (Langmead and Salzberg, 2013). The remaining reads (unmapped) were then assembled *de novo* to obtain contigs using metaSPAdes V 3.9.0 (Nurk et al., 2017) with default settings.

### Virus Identification and Reference Mapping of the Assembled *de novo* Contigs

The resulting *de novo* contigs were compared with other sequences in the National Center for Biotechnology Information (NCBI) GenBank database^1^ (Benson et al., 2012) and the Plant Virus Genome Database (Camacho et al., 2009) using BLASTn search, and the top hit accession was used for virus identification. For each viral species identified, the most frequent annotated accessions in the NCBI was used as reference for alignment and also for the estimation of sequence similarity. The Krona web-based tool (Ondov et al., 2011) was used to visualize BLAST results.

Reference assemblies were performed for complete virus genome sequences by mapping the *de novo* sequences against the most similar existing viral genomes using the read mapping module of CLC genomics workbench version 5.5.1^2^. The sequences were assigned as complete genomes based on comparison with the reference sequences used in the mapping process obtained from BLASTn search results. The *de novo* consensus sequences and consensus sequences from reference mapping were then compared through visual inspection of individual mappings to ensure no artifacts were incorporated as a result of sequencing errors or errors during genome assembly. *De novo* sequences were, however, chosen over the consensus of reference assembly as a precautionary measure in case the viruses identified had considerably diverged from similar viral genome sequences in the GenBank database.

### Validation of Assembled Virus Sequences Through RT-PCR and Sanger Sequencing

The assembled viral sequences were validated through reverse transcription (RT) followed by polymerase chain reaction (PCR) and sent to Macrogen (Europe) for Sanger sequencing. Briefly, viral sequences generated from the Illumima MiSeq were aligned using CLC genomics and consensus sequences used for designing primers using Primer 3. Designed primers were evaluated for specificity using Primer-BLAST (Ye et al., 2012) and tested on the samples in which the viruses had earlier been detected (by NGS). The target viruses were amplified from these samples through RT of total RNA using SuperScript^TM^ III Reverse Transcriptase (Invitrogen^TM^) followed by PCR using the designed primer sequences. Viral amplicons were purified using the QIAquick PCR Purification Kit (Qiagen Inc.), according to manufacturer instructions and quantified using the NanoDrop Spectrophotometer (Thermo Scientific^TM^). The amplicons were sequenced using Macrogen Sanger sequencing. The sequences were trimmed and assembled using the CLC Genomics Workbench version 8.03 with the default settings. The consensus sequences were used for BLASTn search in the NCBI and for comparison with the sequences generated on the Illumina MiSeq System.

### Analysis of Virus Sequences Associated With Papaya Ringspot Disease

The obtained viral sequences from Illumina MiSeq system were used to determine sequence identity (%), open reading frames (ORFs), conserved motifs, and phylogeny. Sequence identities were computed using the Sequence Identity and Similarity (SIAS) tool^3^; the search for ORFs was done using the ORF finder^4^; and conserved protein domains were identified using NCBI conserved domain search program (Marchler-Bauer et al., 2011), whereas the conserved motifs were identified through comparisons with known viral sequences. The sequences from this study and other previously identified viral sequences retrieved from the GenBank database were used to determine phylogenetic relationships among members of the same genus. Briefly, sequences were imported into the CLC Genomics Workbench, aligned and exported in FASTA format, converted to MEGA format, and used for distance and phylogenetic analysis using MEGA 6 software (Tamura et al., 2013). The phylogenetic trees were constructed using the maximum likelihood method based on the JTT matrix (Jones et al., 1992), using 1000 replicates for bootstrap analysis. The recombination detection program (RDP)4 package (Martin et al., 2015) was used to detect recombination in the nucleotide sequences of the identified viruses using RDP (Martin and Rybicki, 2000), GENECONV (Padidam et al., 1999), Bootscan (Martin et al., 2005), MaxChi (Smith, 1992), Chimaera (Posada and Crandall, 2001), 3Seq (Boni et al., 2007), and SiScan (Gibbs et al., 2000) programs implemented in the package with default parameters.

## Results

### Illumina MiSeq Sequencing Statistics

To provide an insight into viruses associated with PRSD symptoms in Kenya, 48 leaf samples were sequenced using the Illumina MiSeq platform. A total of 50,247,269 reads of length between 35 and 151 bp were generated from two runs. The raw reads were filtered to remove those of low quality, leaving a total of 47,800,743 reads with read length ranging from 60 to 151 bp. The number of reads per sample ranged from 465,116 in S1 to 1,809,690 in S43. The GC content ranged from 42 to 48%. Viruses were detected in 38 out of the 48 samples sequenced (Supplementary Table S1).

### Viruses Detected in Symptomatic and Asymptomatic Papaya Leaf Samples

The reads were assembled into 49 contigs, ranging from 469 bases in S11 (collected from Kiambu County) to 10,292 bases in S4 (collected from Nakuru County) (Table 1). The BLASTn search of the *de novo* assembled sequences against the NCBI non-redundant database indicated the presence of Moroccan watermelon mosaic virus (MWMV), cowpea mild mottle virus (CpMMV), and cucumber vein-clearing virus (CuVCV). The BLASTn results of 35 *de novo* assembled sequences from 31 samples shared between 80 and 90% sequence similarities with MWMV genome sequences previously reported in Tunisia, South Africa, Democratic Republic of Congo, and Morocco (GenBank accession numbers: LN810061, EF579955, KU315176 EF211959, and AF305545, respectively). The CuVCV genome sequences were recorded in 11 samples with nucleotide sequence similarities of 72–77% to CuVCV previously reported in Tanzania (GenBank accession number JN591720). Three samples showed sequences closely related to CpMMV and had between 76 and 86% nucleotide sequence similarities to CpMMV sequences reported previously in North America, Brazil, and Ghana (GenBank accession numbers: KC774020, KF554101, and HQ184471, respectively) (Table 1).

**TABLE 1:** Viruses identified in papaya and their sequence similarity (%) with closest homologs in the databases.

**Sample**	**Symptomatic/**	**Symptoms**	**Virus**	**Accessions**		***De novo***	**Similarity**		
**No.**	**asymptomatic**	**expressed^a^**	**identified^b^**	**in NCBI^#^**	**Length**	**coverage**	**(%)**	**Identities**	**E-value**
S1	Symptomatic	WS	MWMV	EF211959	930	2.0	82	766/929	0
S2	Symptomatic	ML, LD, RS	MWMV	KU315176	9769	305.4	80	7726/9645	0
S3	Asymptomatic	–	nd	–	–	–	–	–	–
S4	Symptomatic	ML, RS	MWMV	KU315176	10292	442.6	80	7820/9767	0
S5	Symptomatic	ML, LC	MWMV	EF211959	1010	1.9	87	877/1003	0
S6*	Asymptomatic	–	CpMMV	KF554101	787	2.6	78	331/424	8.00E−65
			MWMV	EF211959	587	1.0	83	356/431	1.00E−101
S7	Asymptomatic	–	nd	–	–	–	–	–	–
S8	Asymptomatic	–	MWMV	KU315176	3229	5.4	83	2668/3223	0
S9	Symptomatic	MO	nd	–	–	–	–	–	–
S10	Symptomatic	MO, LC	MWMV	EF211959	842	4.9	88	741/842	0
S11	Symptomatic	WS	MWMV	AF305545	469	1.2	90	414/462	2.00E−163
S12*	Asymptomatic	–	CuVCV	JN591720	2376	8.5	76	528/697	3.00E−89
			MWMV	LN810061	6337	7.3	81	5164/6348	0
S13	Symptomatic	WS	MWMV	LN810061	9808	400	80	7717/9621	0
S14	Symptomatic	MO	MWMV	LN810061	9772	616	80	7722/9635	0
S15	Asymptomatic	–	MWMV	EF211959	769	2.9	87	627/720	0
S16	Symptomatic	ML, LC	MWMV	EF579955	491	1.6	83	408/489	2.00E−123
S17	Symptomatic	MO	CuVCV	JN591720	9023	324.7	76	1270/1664	0
S18	Symptomatic	MO	nd	–	–	–	–	–	–
S19	Symptomatic	MO, SS, PU	MWMV	LN810061	9804	2239.7	80	7738/9643	0
S20	Symptomatic	ML, WS	MWMV	LN810061	9844	1703.8	80	7728/9636	0
S21	Symptomatic	MO, SS, PU	MWMV	LN810061	9723	3027.0	80	7730/9636	0
S22	Symptomatic	WS, RS	MWMV	KU315176	9706	262.4	80	7727/9624	0
S23*	Symptomatic	MO, PU, VC, LC	MWMV	KU315176	9722	1002.2	80	8197/9763	0
			CuVCV	JN591720	2229	5.0	77	729/949	8.00E−144
S24	Symptomatic	ML, WS	MWMV	LN810061	9861	1723.4	80	7719/9636	0
S25	Symptomatic	ML	CuVCV	JN591720	9081	3155.4	75	1220/1637	0
S26*	Asymptomatic	–	MWMV	LN810061	2004	1.4	84	1688/2000	0
			CuVCV	JN591720	5596	63.9	73	1077/1468	9.00E−142
S27	Symptomatic	ML, PU	MWMV	LN810061	9772	767.0	80	7728/9637	0
S28	Asymptomatic	–	nd	–	–	–	–	–	–
S29*	Asymptomatic	–	CuVCV	JN591720	9079	567.4	72	1124/1554	1.00E−122
			MWMV	EF579955	2011	1.4	81	1629/2006	0
S30	Asymptomatic	–	nd	–	–	–	–	–	–
S31	Symptomatic	PU, LC	MWMV	LN810061	9792	2454.9	80	7724/9636	0
S32	Asymptomatic	-	nd	–	–	–	–	–	–
S33*	Symptomatic	MO, SS, VC	MWMV	LN810061	9717	612.7	80	7727/9634	0
			CuVCV	JN591720	9080	2192.4	73	1072/1466	2.00E−136
S34*	Symptomatic	MO	CuVCV	JN591720	9069	891.9	73	1074/1466	7.00E−140
			CpMMV	HQ184471	3204	5830	76	507/668	3.00E−85
			MWMV	EF211959	1433	5.0	85	1222/1432	0
S35	Symptomatic	RS	MWMV	KU315176	9675	84.7	80	7708/9610	0
S36	Symptomatic	RS	MWMV	KU315176	9726	1252.6	80	7726/9639	0
S37	Asymptomatic	–	nd	–	–	–	–	–	–
S38	Asymptomatic	–	nd	–	–	–	–	–	–
S39*	Symptomatic	ML	CpMMV	KC774019	8196	532.5	86	7046/8217	0
			CuVCV	JN591720	9028	74.2	75	611/813	1.00E−98
S40	Symptomatic	ML, LC	MWMV	LN810061	9722	921	80	7700/9637	0
S41	Symptomatic	MO, SS, RS	MWMV	LN810061	9677	27.5	80	7705/9598	0
S42	Symptomatic	MO, VC	MWMV	LN810061	9734	2454.4	80	7732/9635	0
S43*	Symptomatic	VC, ML	MWMV	KU315176	10221	1129.8	80	7805/9747	0
			CuVCV	JN591720	9072	3740.8	73	1065/1462	2.00E−131
S44	Symptomatic	MO, SS, PU	MWMV	KU315176	9747	742.0	80	7818/9643	0
S45*	Symptomatic	ML, PU	MWMV	KU315176	9723	1106.2	80	7732/9653	0
			CuVCV	JN591720	9080	1001	73	1073/1469	7.00E−135
S46	Symptomatic	MO, SS, PU	MWMV	LN810061	9786	5911	80	7735/9643	0
S47	Symptomatic	MO, SS, PU	MWMV	LN810061	1556	3.0	82	1254/1528	0
S48	Asymptomatic	–	nd	–	–	–	–	–	–

Key: *Indicates co-infections; nd, not detected; ^*a*^symptom description; WS: water-soaked marks on the stem/petioles; ML: Mottling; MO: Mosaic; LD: leaf distortion; LC: leaf curl; SS: shoe stringing of leaves; PU: puckering; VC: vein clearing; and RS: ringspots on the fruits; ^*b*^virus identified; MWMV: Moroccan watermelon mosaic virus, CuVCV: Cucumber vein-clearing virus and CpMMV: Cowpea mild mottle virus; ^#^BLASTn search showing only topmost accession hit.

Cases of single and co-infections of the viruses obtained in this study were also observed in the samples. Single virus infections of MWMV were detected in 26 samples collected from Makueni, Nakuru, Homabay, Taita Taveta, Kiambu, Busia, Kilifi, Murang’a, Kirinyaga, Embu, Meru, and Machakos counties, whereas single CuVCV infection was found in two samples collected from Makueni and Kwale (Table 1). The co-infections of MWMV and CuVCV were the majority and were detected in seven samples collected from Kitui, Embu, Machakos, Tharaka Nithi, Meru, and Makueni counties. The existence of mixed infections of MWMV and CpMMV, CuVCV, and CpMMV, as well as MWMV, CpMMV, and CuVCV were reported in one sample each from Baringo, Kitui, and Meru counties, respectively. Due to the misleading interpretations of partial sequences for virus identity (Jo et al., 2017), we used complete genome sequences of the viruses identified through BLASTn search in subsequent analysis. In the GenBank database, the complete genome of MWMV is about 9.7 kbp and CpMMV is 8.1 kbp, while the CuVCV genome sequence is partial (5218 bp). However, we were able to designate sequences of CuVCV as complete or near-complete genomes based on the information of genome features in the species *Carlavirus* in the database. Out of the 22 counties surveyed, 22 complete virus genome sequences detected in 11 counties—namely, Homabay, Kisumu, Busia, Kiambu, Meru, Embu, Kirinyaga, Murang’a, Machakos, Makueni, and Nakuru—were closely related to MWMV, the most prevalent virus in the samples analyzed (Table 1). The CuVCV was the second most prevalent, and eight complete viral genome sequences were detected in samples collected from Makueni, Kwale, Tharaka Nithi, Meru, Kitui, and Machakos counties. Only one complete genome of CpMMV with 8196 bases was detected in a sample collected from Kitui County (Table 1).

### The Relationship Between Virus Detection and Disease

**FIGURE 2:**
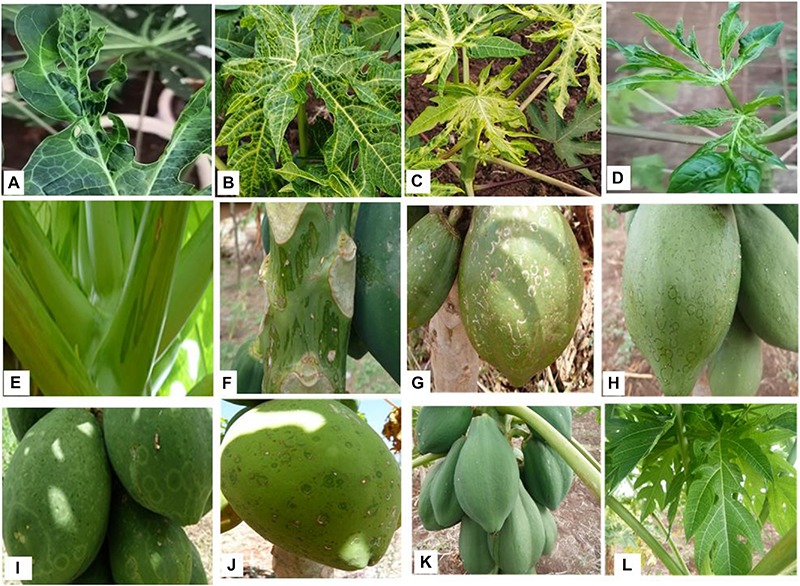
Diversity in PRSD symptoms observed during the field survey. **(A)** Puckering. **(B)** Mottling. **(C)** Mosaic. **(D)** Leaf deformation. **(E)** Oily streaked petioles. **(F)** Oily streaked stem. **(G)** Necrotic rings. **(H,I)** Circular rings. **(J)** Concentric water soaked lesions. **(K)** Asymptomatic fruits. **(L)** Asymptomatic leaves.

The symptoms observed on infected leaves varied significantly and ranged from mottling, mosaic, shoe–stringing, curling, and puckering. Similarly, on fruits, symptoms such as concentric water-soaked lesions, circular ringspots, and necrotic rings were observed. On the upper part of the stem and leaf petioles, numerous water-soaked lesions were seen (Table 1 and Figure 2). There was a clear association between virus presence and symptoms in papaya plants. Hence, the majority of the symptomatic plants (32 out of 34) tested positive for viral infection. There were, however, cases in which plants were asymptomatic but viruses were detected through NGS (six out of 14) (Table 1). More interestingly, in cases where fruits exhibited concentric water-soaked lesions, circular ringspots, and necrotic rings, single infections with MWMV were detected through NGS, while in occurrences of single infection of CuVCV, mottling was observed. Co-infections of MWMV and CuVCV were found in plants that were either asymptomatic or in those exhibiting mottling, mosaic, vein clearing, or puckering symptoms. Co-infection of CuVCV with CpMMV was detected by NGS in plants exhibiting mottling symptoms. To exclude the presence of PRSV and to confirm that MWMV was associated with ringspots, the spots were excised from the infected fruits and the viruses concentrated using protocol described by Blomström et al. (2010). RNA was extracted using TRIzol LS Reagent (Invitrogen) and further purified using RNeasy Mini Kit columns (Qiagen). The RNA was sequenced using Illumina Miseq system and a *de novo* assembled 9700 bp sequence was obtained that shared an 80% sequence similarity with MWMV sequences in GenBank. This strongly suggested that MWMV is associated with the symptoms and is the putative cause of the ringspots on papaya fruits in Kenya.

### RT-PCR and Sanger Sequencing Validation of Viruses

Sanger sequencing of the viral amplicons obtained from RT-PCR yielded sequences that were 100% identical to those generated *de novo* from assembled Illumina sequences, confirming that the *de novo* assembly gave accurate sequences of the viruses’ genomes. Primers designed, their sequences, the target region in the genome, and the expected size of the virus amplicon are shown in Table 2. To further authenticate the association of MWMV with ringspot symptoms on fruits, extracts from ringed spots were tested using RT-PCR approach in few samples among the 48 sequenced, indicating a strong association of the virus with ringspots on the fruits (Supplementary Figure S1).

**TABLE 2:** Primers, target region of virus genome, and expected amplicon size.

			**Amplicon**
**Virus**	**Genome region**	**Primer sequence**	**size (bp)**
MWMV	Coat protein	ATCATCGCAGAACCAAGGCA	697
		ATCAACAGTGTGCCTCTCCG	
	Cylindrical Inclusion	TCTCAGCTAGCACGCAACAA	315
		CGGTGTTGAGCCAAACGAAG	
CuVCV	Coat protein	AGACCAAAGAGTGCTTCGGG	304
		TAGGAACTCCCAGTCCCTCG	
	RNA dependent RNA polymerase (RdRp)	AGTGGTTGCGAGTTGTTCCA	420
		CAACCAAAGTCCCCATCCGA	
CpMMV	Coat protein	AACATGGCGACAGCTGAAGA	694
		GAAGAGCGACCAGTTCCCAA	
	RdRp	CGGCTCAAAGTATTGCCTGC	761
		TGTTCTGACGCAGCCGTAAT	

### Genome Organization of MWMV in This Study and Determination of Its Phylogenetic Affinities

The obtained viral genome sequences of MWMV were deposited in GenBank under accession numbers MH595736 through MH595746. The genomes are 9712–9725 nucleotides (nt) long organized into 142–155 non-coding nt at their 5′ terminus, followed by 9375 nt encoding the polyprotein, from which all the proteins of the virus are derived, and 194–197 non-coding nt at 3′ terminus. The polyprotein codes for 3124 amino acids (aa) with a molecular weight of between 353.9 and 354.5 kDa. The base composition includes 31.4–31.6% adenine, 18.8–19.0% cytosine, 23.3–23.4% uracil, and 26.1–26.4% guanine.

The genomes are single-stranded positive-sense RNA virus with a single ORF that is translated into a single large polyprotein. The polyprotein has nine putative cleavage sites, yielding 10 functional proteins. The length (nt and aa) and organization of the 10 proteins are as follows: P1 (1035/232), helper component protease (Hc-Pro) (1371/457), P3 (1041/347), 6K1 (156/52), cylindrical inclusion (CI) (1905/635), 6K2 (171/57), VPg (570/190), nuclear inclusion a (NIa) (717/239), nuclear inclusion b (NIb) (1550/517), and coat protein (CP) (855/285).

Several conserved motifs found in potyviruses were identified in the MWMV genomes detected in this study. In the HC-Pro gene there are highly conserved “RITC,” “CSC,” and “PTR” motifs, which are associated with virus transmission (Huet et al., 1994; Blanc et al., 1998) and “FRNK(X)_12_CDN” that is involved in symptom development (Gal-On, 2000). The RNA helicase function motifs “GAVGSGKST” and “PTR” were found to be conserved in the N-terminal region of the CI. Three RdRp motifs “YCDADGS,” “GNNSGQPSTVVDNTLMV,” and “NGDDL” responsible for potyviral genome replication (Hong and Hunt, 1996) were present in NIb. The well-characterized DAG motif, highly conserved among all aphid transmissible *potyviruses* (Atreya et al., 1995) was found in the N-terminus of the CP. A stretch of glutamic acid and lysine repeats (KE repeats) was found after the “DAG” motif in the N-terminus of the CP in all MWMV signatures in this study.

*Potyviruses* are usually classified based on the percentage of sequence identity in the polyprotein or CP. Viruses sharing more than 75% nt and 80% aa sequence identity in the CP or polyprotein are considered the same species (Adams et al., 2005). The MWMV viruses from this study share 98.1–98.6% aa (97.5–98.5% nt) sequence identity among themselves in the polyprotein region. They also share 89.8–90.0% aa (79.2–79.6% nt) with isolate from Tunisia (GenBank accession number EF579955), and 90.3–90.5% aa (79.6–79.7% nt) with isolate from South Africa (GenBank accession KU315176) in the polyprotein. Furthermore, sequence identity of 93.7–95.1% aa (84.3–84.9% nt) and 94.1–95.1% aa (83.9–85.3% nt) with Tunisian and South African MWMV isolates in the CP was observed, respectively (Table 3).

**TABLE 3:** Nucleotide and amino acid sequence identities (%) between Kenyan MWMV isolates and those from Tunisia and South Africa.

	**Among Kenyan isolates**		**Between Kenyan and a Tunisian**		**Between Kenyan and a South**	
**Genome features**	**(MH595736-46)**		**isolate (EF579955)**		**African isolate (KU315176)**	
	**aa**	**nt**	**aa**	**nt**	**aa**	**nt**
Polyprotein	98.1–98.6	97.5–98.5	89.8–90.0	79.2–79.6	90.3–90.5	79.6–79.7
P1	94.2–96.5	96.4–97.8	65.1–68.1	69.4–70.5	67.8–69.0	70.3–71.3
HC-Pro	97.4–98.9	97.0–98.8	93.7–94.7	79.7–80.3	93.7–94.7	79.5–80.4
P3	97.9–99.3	97.5–98.8	87.4–88.5	79.3–79.9	89.2–90.1	79.9–80.7
6K1	100	96.8–99.4	94.2	80.8–81.4	94.2	82.1–83.3
CI	98.9–99.7	97.8–98.6	95.3–95.7	80.9–81.7	95.7–96.4	80.9–81.3
6K2	98.2–100	96.5–99.4	86.0–87.7	74.9–76.6	89.4–91.2	75.4–77.2
VPg	97.9–100	97.4–99.3	83.6–85.2	78.1–79.1	83.7–84.7	75.1–76.1
NIa	98.7–99.6	97.4–98.7	92.1–92.5	78.9–79.5	92.5–93.7	79.9–80.6
NIb	98.6–99.4	97.5–98.6	94.4–95.0	80.5–81.1	94.2–94.8	81.2–82.1
CP	97.6–99.7	97.6–99.2	93.7–95.1	84.3–84.9	94.1–95.1	83.9–85.3

**TABLE 4:** Viruses used in phylogenetic analysis, host plants, country of origin, and GenBank accession numbers.

**Virus**	**Host**	**Country**	**Accession No.**
Moroccan watermelon mosaic virus	Zucchini	Tunisia	YP_001552410
Moroccan watermelon mosaic virus	Watermelon	Greece	CEO86971
Moroccan watermelon mosaic virus	PattyPan squash	South Africa	ANF99508
Moroccan watermelon mosaic virus	Pumpkin	South Africa	ANF99506
Moroccan watermelon mosaic virus	Papaya	Kenya	MH595736-46
Sudan watermelon mosaic virus	Snake cucumber	Sudan	YP_009407951
Zucchini shoestring virus	Pumpkin	South Africa	ANH22633
Algerian watermelon mosaic virus	Squash	Algeria	YP_001931956
Papaya ringspot virus	Pumpkin	South Korea	ARR28779
Papaya ringspot virus W	Cucurbit	East Timor	ASK05490
Papaya ringspot virus Y	*Caica papaya* L.	Taiwan	AFU56872
Leek yellow stripe virus	Garlic	Japan	BAE72834
Cucumber vein-clearing virus	Watermelon	Tanzania	AEP83730 AEP83732
Cowpea mild mottle virus	*Carica papaya* L.	Kenya	MK984605
Cowpea mild mottle virus	*Vigna unguiculata*	Ghana	YP_004035878
Cowpea mild mottle virus	*Vigna unguiculata*	India	ATV94962
Cowpea mild mottle virus	*Phaseolus vulgaris*	United States	AHG23050
Cowpea mild mottle virus	*Glycine max*	Brazil	AGS13088 AGS13092
Cowpea mild mottle virus	*Glycine max*	Brazil	AGS13112
Cowpea mild mottle virus	*Glycine max*	Brazil	AGS13100
Papaya mottle virus	*Carica papaya* L.	Kenya	MK984599 MK984600 MK984601 MK984603 MK984604
Papaya mild mottle virus	*Carica papaya* L.	Kenya	MK984597 MK984598 MK984602
Pepper virus A	*Capsicum annum*	India	YP_009357230
Pepper virus A	*Capsicum annum*	India	AOY34821
Jasmine virus C	*Jasminum sambac*	Taiwan	YP_009275350
Potato virus M	Potato	Iran	AGS32289
Potato virus M	Potato	Germany	ACF05251
Cowpea mild mottle virus	Cowpea	Puerto Rico	ACZ71185
Cowpea mild mottle virus	Cowpea	Taiwan	AFO84257
Cowpea mild mottle virus	Urdbean	India	AJE62535
Cowpea mild mottle virus	*Glycine max*	India	AGH18394
Cowpea mild mottle virus	*Arachis hypogea*	India	AAB94082
Cowpea mild mottle virus	*Arachis hypogea*	India	AAB94083
Cowpea mild mottle virus	Mungbean	India	AJE62533
Pepper virus A	*Capsicum annum*	Taiwan	YP_009357233
Hippeastrum latent virus	*Hippeastrum hybridum Hort*	Taiwan	YP_002308451
Potato virus M	Potato	Bangladesh	ATG34149
Phlox virus M	Phlox	United States	ACI06093
Phlox virus M	Phlox	United States	ABP68910

**FIGURE 3:**
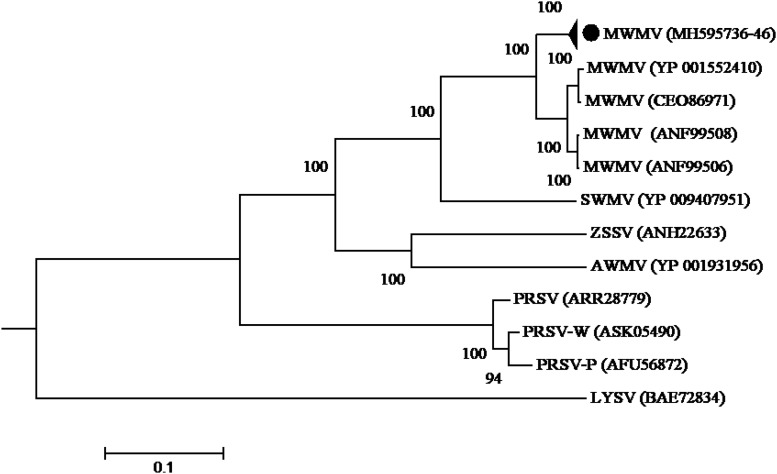
Phylogenetic relationships among MWMV isolates and closely related potyviruses. The phylogenetic tree was generated using the maximum likelihood method based on JTT matrix-based model. The sequence generated in this study is shown by black circle. The viruses, host plant, country of origin, and GenBank accession numbers used in phylogenetic tree are shown in Table 4.

Phylogenetic analysis built using the complete polyprotein aa sequences showed that all the MWMV isolates detected in this study formed a single cluster separate from South Africa, Tunisia, and Greece sequences. A clear geographical clustering was also observed. Isolates from South Africa clustered separately from the Mediterranean ones. The PRSV that was previously believed to be responsible for the ringspot symptoms showed a distinct evolutionary pathway from MWMV as depicted in the phylogenetic tree (Figure 3).

### Cowpea Mild Mottle Virus: Identification and Phylogeny

The complete genome sequence of the CpMMV identified in this study is 8151 nt long, excluding the poly-A tail (GenBank accession number MK984605). The genome is single-stranded, positive-sense with six ORFs. The ORF1 encodes a RdRp gene consisting of 1859 aa with an estimated molecular weight of 211.3 kDa and four conserved motifs, including viral methyltransferase (Rozanov et al., 1992), RdRp (Koonin, 1991), *Carlavirus* endopeptidase (Peptidase C23) (Lawrence et al., 1995), and viral (superfamily 1) RNA helicase (Viral_helicase1) (Gorbalenya and Koonin, 1989). The ORFs 2, 3, and 4 encode the triple gene block proteins (TGB1-3p, with molecular weights of 25.8, 11.6, and 7.6 kDa, respectively), that are essential for virus movement. The ORF 5 encodes the CP comprising of 288 aa with a molecular weight of 32 kDa and contains a strong conserved motif “His-X8Asp-X15Thr-Gly-Gly” at aa position 246–273 in the C-terminal region of the CP (Naidu et al., 1998). The ORF 6 encodes a cysteine-rich protein (CRP) with nucleic acid-binding protein (NaBP) consisting of 109 aa with a molecular mass of 12.3 kDa.

**FIGURE 4:**
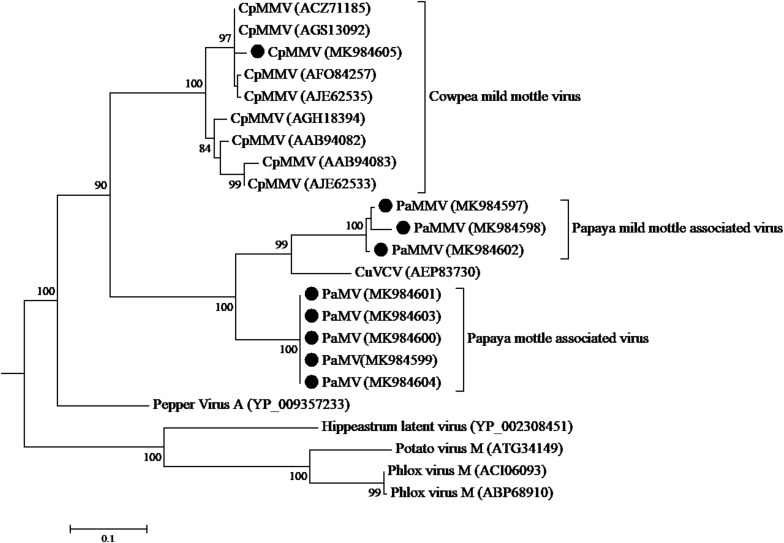
Phylogenetic analysis of coat protein amino acid sequences among CpMMV, PaMMV, PaMV, and closely related *Carlavirus* generated using maximum likelihood method based on JTT matrix-based model. The sequences generated in this study are shown by black circles. The viruses, host plant, country of origin, and GenBank accession numbers used in phylogenetic tree are shown in Table 4.

**FIGURE 5:**
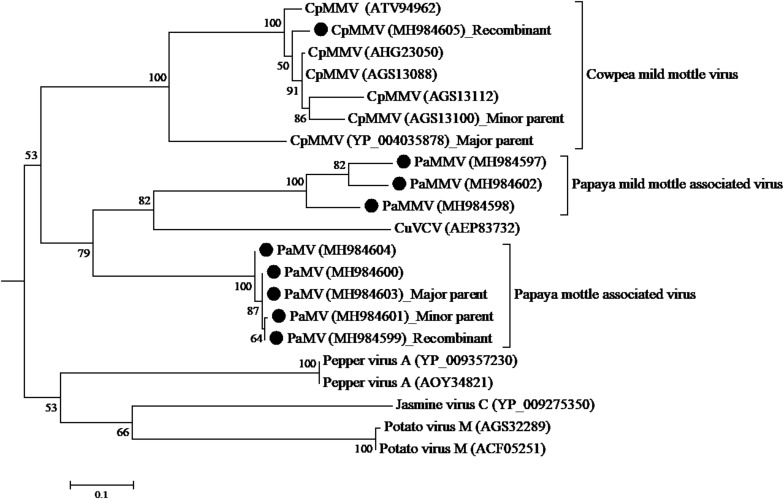
Phylogenetic analysis of RdRp amino acid sequences among CpMMV, PaMMV, PaMV, and closely related *Carlavirus* generated using maximum likelihood method based on JTT matrix-based model. The sequences in this study are shown with black circles. The viruses, host plant, country of origin, and GenBank accession numbers used in phylogenetic tree are shown in Table 4.

The CP sequence comparison of CpMMV isolate from this study with sequences in the database, indicated that the Kenyan CpMMV shares 84.7, 84, and 82.6% aa sequence identities with Brazilian (GenBank accession number AGS13100), Ghanaian (GenBank accession number YP-004035878), and Indian (GenBank accession number ATV94962) isolates, respectively. However, in the RdRp gene, the Kenyan CpMMV isolate shares 90.7, 88.6, and 72% aa sequence identities with the Indian, Brazilian and Ghanaian isolates, respectively. CpMMV sequences in this study clustered together with those of several other CpMMV isolates from the GenBank with a strong bootstrap support of 100% based on either CP or RdRp gene (Figures 4, 5).

Analysis of the ORF1 nucleotide sequences using seven different algorithms showed evidence of recombination. A recombination was detected with major parent being a Ghanaian isolate (YP-004035878) at positions 1–34 and 5330–5650; and the minor parent being a Brazilian isolate (AGS13100) at positions 35–5329. The recombination was detected by four programs; MaxChi Chimaera, Siscan, and 3Seq with a *P*-value of 1.893 E-06. No recombination was detected in the other genes.

### Molecular Characterization of Putative *Carlaviruses* Identified Papaya

The genome sequences of the putative *Carlavirus* (PaMV and PaMMV) are linear, single-stranded positive-sense RNA viruses with a poly-A tail consisting of six ORFs, encoding the following proteins; RdRp, movement proteins (i.e., triple gene block, CP, and CRP with NaBP with arrangement typical of the genus *Carlavirus*) (Martelli et al., 2007; Adams et al., 2012). A BLAST search using individual ORF sequences revealed that these sequences belong to the genus *Carlavirus* in the family *Betaflexiviridae*. Functional protein domains known to be conserved in *Carlaviruses* were also detected in isolates from this study. In the ORF1, there are viral RNA methyltransferase (Vmethyltransf), RdRp_2 Superfamily, *Carlavirus* endopeptidase (Peptidase_C23), and Viral (Superfamily 1) RNA helicase (Viral_helicase1). The RdRp domain also contains the characteristic core motif SGX_3_TX_2_NT_22_GDD found in *Carlaviruses* (Martelli et al., 2007; Adams et al., 2012), while the CP has the conserved CP domain of carlviruses Flexi_CP_N and Flexi_CP.

These viruses were found in a mixed infection with MWMV or CpMMV. The association of symptoms to them hence becomes problematic. However, in cases where these viruses were identified as a single infection through NGS, severe and mild mottling symptoms were observed. Accordingly, we have proposed naming of these viruses papaya mottle-associated virus (PaMV) and papaya mild mottle-associated virus (PaMMV).

The PaMV detected in this study is 9061–9071 nt long, excluding the poly-A tail (GenBank accession numbers MK984599, MK984600, MK984601, MK984603, and MK984604) and were obtained from samples collected from Machakos, Meru, Tharaka Nithi, and Makueni counties. The size of the ORFs is as follows: ORF1 1558 aa (partial) for MK984600 and 2162 aa (complete) (175.1–248 kDa), ORF2 228 aa (25.3 kDa), ORF3 108 aa (11.6 kDa), ORF4 63 aa (6.9 kDa) ORF5 276 aa (30.8 kDa), and ORF 6 102 aa (11.6 kDa). The PaMMV, on the other hand, was identified in three samples collected from Makeuni, Kwale, and Kitui counties (GenBank accession numbers MK984597, MK984598, and MK984602). The genomes are 9028-, 9023-, and 9070-nt long, respectively (excluding the poly-A tail). The ORF1 comprises of 2154 aa (245 kDa); ORF2 228 aa (25.3 kDa); ORF3 108 aa (11.6 kDa); ORF4 75 aa (8.1 kDa); ORF5 288 aa (32.1 kDa); and ORF6 103 aa (11.9 kDa).

A BLASTn search using PaMV and PaMMV genomes as query sequences in the GenBank always returned CuVCV as the most similar sequence. However, close analysis indicated that there were differences in the size of ORF1, ORF4, ORF5, and ORF6 between the two sets of viruses. The ORF1 in PaMV comprises 2162 aa, whereas PaMMV has 2154 aa. The ORF4 consists of 63 and 75 aa; the ORF5 276 and 288 aa; and the ORF6 102 and 103 aa for PaMV and PaMMV, respectively. There were insertions or deletions (indels) of aa sequences observed on these ORFs when sequence alignment was performed, contributing to differences in the sizes of these ORFs. These indels are unlikely to be due to sequencing error because they were also found in other published *Carlaviruses*. Furthermore, these variations were mapped to a common area in the samples analyzed (as was the case on the 5′ end of the ORFs).

Based on the species demarcation criterion of 72% nt and 80% aa similarity in the CP or RdRp among *Carlviruses* (Adams et al., 2012), it is clear that the two viruses could be considered distinct species within the genus *Carlavirus*. The percentage of sequence identity indicated that the isolates in this study shared 75.7–78.1% aa and 63.6–67.6% nt sequence identities in the CP gene with CuVCV isolate from Tanzania (GenBank accession number AEP83730) (Table 5), values below the threshold for species discrimination in *Carlviruses*. Additionally, PaMV and PaMMV shared less than 80% aa and 75% nt sequence identities in the CP, thereby qualifying them to be different species of the same *Carlavirus* genus.

**TABLE 5:** Percentage sequence identities in the coat protein of PaMV and PaMMV *Carlaviruses* with the closest homolog CuVCV from Tanzania (AEP83730).

		**PAMMV**			**PaMV**			**CuVCV**		
	**GenBank**									
	**Acc. No.**	**1**	**2**	**3**	**4**	**5**	**6**	**7**	**8**	**9**
PaMMV	1. MK984597	–	92.0	93.4	75	75	75	75	75	77.4
	2. MK984598	**73.2**	–	91.7	72.9	72.9	72.9	72.9	72.9	76.7
	3. MK984602	**71.2**	**74.1**	–	74.7	74.7	74.9	74.7	74.6	78.1
PaMV	4. MK984599	**64.6**	**62.9**	**63.5**	–	100	100	100	100	75.7
	5. MK984600	**65.7**	**64.5**	**64.4**	**77.3**	–	100	100	100	75.7
	6. MH984601	**66.7**	**63.4**	**64.2**	**75.4**	**74.6**	–	100	100	75.7
	7. MK984603	**66.3**	**63.2**	**65.4**	**72.3**	**76.9**	**75.7**	–	100	75.7
	8. MK984604	**66.8**	**63.7**	**64.9**	**73.6**	**75.5**	**75**	**76.7**	–	75.7
CuVCV	9. AEP83730	**66.4**	**65.3**	**66**	**65.8**	**65.9**	**63.6**	**67**	**67.6**	–

*Percentage nucleotide sequence identity in coat protein (bold numbers) and amino acids (regular text).*

The phylogenetic trees generated using the CP and the RNA-dependent RNA polymerase gene (Figures 4, 5, respectively) support the proposed species classification within the genus *Carlavirus*. The PaMV isolates formed a monophyletic group, whereas PaMMV isolates clustered together, closer to the CuVCV isolate from Tanzania (AEP83730).

A recombination event was detected in PaMV isolate MK984599, collected from Tharaka Nithi county—with the major parent being MK984603 from Machakos County (at positions 1–1169 and 2280–6806) and the minor parent being MK984601 from Meru County (at positions 1170–2279) (Figure 5). The recombination was supported by four programs: MaxChi, Chimaera, Siscan, and 3Seq, with a *P*-value of 4.718 E-06. No recombination was detected in the coat protein gene.

## Discussion

Through Illumina MiSeq sequencing, we characterized complete genome sequences of MWMV, a *Potyvirus*; CpMMV, a *Carlavirus*, and two novel yet divergent *Carlaviruses* (namely, PaMV and PAMMV) in symptomatic and asymptomatic papaya leaves collected from Kenyan fields. This study provides the first report of these viruses in papaya in Kenya. We also report for the first time the infection of papaya with CpMMV, PaMMV, and PaMV. The presence of MWMV in papaya crops in Kenya would suggest that either the virus is increasing in its geographical distribution or that it has been present in papaya and/or in other host plants but has previously gone undetected. The study also suggests the emergence of new viruses (CpMMV, PaMMV, and PaMV)—or that the viruses have been present but have recently moved to papaya from other hosts—that are now posing a serious threat to papaya production in the country. The sequencing strategy used in this study targeted viruses with poly-A tail, as per the TruSeq RNA Illumina protocol used. The possibility of new or additional viruses falling outside of this detection approach possibly infecting papaya crop cannot be ruled out. Additional viral metagenomics studies could help in elucidating the complete diversity of viruses that are infecting papaya in Kenya.

The characterization of a plant virus disease with a known etiology usually relies on the symptoms expressed in the host plants because they are easy to recognize, especially if they are disease-specific. Symptoms also aid in roguing of diseased plants as a strategy for preventing virus spread (Naidu and Hughes, 2003). Potyviruses have limited host ranges and can be identified based on the characteristic symptoms they produce in certain host plants (Shukla and Ward, 1989). In this study, the symptoms observed on papaya plants included those that are attributed to PRSV infection (Tripathi et al., 2008; Zhao et al., 2016), although PRSV was not detected in our samples. This explains why earlier attempts to detect PRSV in diseased plants through ELISA and RT-PCR procedures using primers specific to PRSV (Ombwara et al., 2014) were unsuccessful. Failure to detect PRSV in papaya plants exhibiting the above symptoms shows the limitations of using symptoms for disease diagnosis (Candresse et al., 2014). Although we did not complete Koch’s postulates for the identified viruses, results from NGS and RT-PCR established a strong association between MWMV and the ringspot symptoms we observed in Kenyan papaya.

Establishing associations between a specific viral infection and the symptoms expressed in host plants can be further complicated by mixed viral infections (Marais et al., 2015). For instance, in the co-infection of papaya with MWMV and PaMV, we were not able to associate specific symptoms to either virus. However, Illumina sequencing of RNA extracted from the ringed spots of fruits strongly suggested MWMV to be associated with the ringspots on Kenyan papaya. This virus has also been reported in papaya plants exhibiting ringspots in Congo (Arocha et al., 2008). However, papaya plants showing ringspot symptoms could also be infected with other viruses such as PaMV, PaMMV, or CpMMV, as observed in this study. The occurrence of mixed viral infections in papaya has also been reported in Mexico (Noa-Carrazana et al., 2006). In many samples sequenced in this study, MWMV was found with other viruses, suggesting that co-infections of these viruses in papaya plants is not an uncommon phenomenon. The co-infection of MWMV with CuVCV has also been reported in watermelon in Tanzania (Menzel et al., 2011). Plant viruses co-infecting the same host may generally interact in either a synergistic or antagonistic way (Syller, 2012). Whether this is the case for viruses present in the papaya in Kenya remains to be determined.

The clear association between virus presence and symptoms expression in papaya plants was clearly observed with the majority of the symptomatic plants testing positive for virus(es) infection. Some of our samples, despite not having clear visual viral symptoms showed the presence of virus(es) when sequenced on the Illumina MiSeq platform. Most of these viral sequences from asymptomatic leaves were partial except in one instance in Tharaka Nithi County, where we recovered complete viral genome of PaMV. Several factors may have contributed to the absence of symptoms in these infected samples, including papaya variety or cultivar, plant age and number of days post-infection at the time samples were collected (Singh and Shukla, 2011) and virus titers (Ghoshal and Sanfaçon, 2015). Further, masking of symptoms occurs in virus infected papaya plants depending on the environmental conditions during the season (Kabir et al., 2017). Thorough screening of asymptomatic plants is therefore paramount for better disease management.

Since the discovery of MWMV in papaya in Congo a decade ago (Arocha et al., 2008), the virus has not been reported again in papaya. This work represents the second report of MWMV in papaya worldwide. MWMV has been reported in Africa and the Mediterranean region mostly in cucurbits such as *Cucurbita pepo* (Yakoubi et al., 2008; Ibaba et al., 2016; Kidanemariam et al., 2019), *Cucumis melo* (Lecoq et al., 2001), *Lagenaria bleviflora*, and *Adenopus breviflorus* (Owolabi et al., 2012; Mofunanya and Edu, 2015). Our findings would suggest that papaya is an additional natural host for MWMV and that there could be more wild or cultivated hosts requiring further determination.

Phylogenetic analysis of MWMV revealed a clear geographical clustering pattern showing the Kenyan isolates on one clade and the South African and Mediterranean ones in separate groups. Similar geographical grouping of MWMV isolates was also reported in Tunisia (Yakoubi et al., 2008). If this clustering based on host or geographic origin remains to be determined. Nevertheless, all MWMV isolates in this study show high sequence identity values, despite their different counties of origin suggesting a recent introduction in the country.

Recombination and mutation events are major forces attributed to evolution in plant viruses and are associated to adaptation to new hosts, often leading to emergence of new variants and resistance breaking strains (Ohshima et al., 2002; García-Arenal et al., 2003; Nagy, 2008; Desbiez et al., 2011; Kwak et al., 2015; Xie et al., 2016). BLASTn search of PaMV and PaMMV sequences showed CuVCV to be their closest species genetically. Similarly, these viruses seem to have a common ancestor (from the phylogenetic analysis). Moreover, recombination events were detected in PaMV sequences within the RdRp genes although these recombination events did not change the phylogenetic groupings of the isolates. Further comparison between PaMV and PaMMV ORFs showed several indels. From the results, it is likely that these two viruses evolved from a common ancestor. However, a detailed analysis of these viruses from different hosts and locations will be critical in elucidating their evolutionary paths and for determining if these events have any biological significance such as host range and virulence. The CpMMV under this study is a recombinant between Ghanaian (YP_004035878) and Brazilian (AGS13100) isolates. In the phylogenetic tree, it clustered away from both parents. This result points to the likelihood that CpMMV from Kenya is a separate strain from its parents, which could have been caused by recombination event.

The detection of single or co-infections of *potyviruses* and *carlaviruses* associated with PRSD in the papaya fruit crop in Kenya in both symptomatic and asymptomatic samples are causes for concern. These viruses cause symptoms resembling other viral diseases and could escape routine detection, thus resulting in a considerable reduction in fruit yield and quality. The inability to recognize a symptomless plant harboring a virus could also result in inadvertent exposure of other crops in the country to a potential inoculum source. Although the insect vectors transmitting these viruses and the mode of transmission in papaya are yet to be established, the MWMV in papaya is probably transmitted by aphids, while CpMMV could be vectored by whiteflies (Naidu et al., 1998). Because of the close relationship between PaMMV and PaMV with the white-fly transmitted cucumber vein-clearing virus, there is a likelihood that they are also transmitted by whiteflies (Menzel et al., 2011). Papaya in Kenya is propagated by seeds and the possibility that any of these viruses is seed transmitted cannot be ruled out. However, further studies are needed to identify specific insect vectors and examine the likelihood of virus transmission to papaya in Kenya and their likely wild hosts of these viruses.

As the viruses identified continue to impact negatively on the livelihoods of many farming householders, there is an urgent need to develop an integrated management strategy for the different virus diseases. Current management practices include the use of chemicals to control the insect vectors and to reduce their populations and limit the spread of viruses, and also by rouging diseased plants which act as sources of viral inoculum. However, these measures are mostly not effective. A larger percentage of farmers also do not employ any control measures (Mumo et al., 2019, unpublished data). Papaya cultivars resistant to the pathogens or less attractive to the insect vectors are also not available to the poor farmers. Therefore, promoting the use of virus-free seeds and seedlings and implementing quarantine measures could help prevent the spread of these viruses to areas that are currently virus-free.

Further, virus-specific primers developed in the current study will help to regularly monitor both symptomatic and asymptomatic plants where necessary and discover new infections. These will help prevent future spread of the viruses as well as developing ways of combating and reducing their effects on papaya crops. However, other management and control options, such as identification of tolerant germplasm as well as alternative hosts, need to be explored.

## Conclusion

Two viruses, namely, MWMV and CpMMV, in addition to two newly discovered viruses infecting papaya, tentatively named PaMV and PaMMV, have been discovered in this study and are associated with PRSD in Kenya. Given the rate at which papaya planting materials are exchanged between farmers in different counties in Kenya (Asudi, 2010), it is likely that these viruses, although currently restricted in specific counties, could quickly spread to other papaya-growing areas in the region. Additional and extensive surveys should be carried out to determine the prevalence and distribution of these viruses in adjacent areas to develop and establish control and management measures to prevent further spread. However, further studies are needed for complete classification of PaMV and PaMMV and to understand the risk they pose to papaya and other crops in the region.

## Data Availability Statement

RNAseq raw data are available at NCBI, Bioproject ID: PRJNA595878. Complete genomes were deposited to NCBI under the following GenBank Accession Numbers: MWMV (MH595736-MH595746), CpMMV (MK984605), PaMV (MK984599, MK984600, MK984601, MK984603, and MK984604), and PaMMV (MK984597, MK984598, and MK984602).

## Author Contributions

NM, GM, EA, FR, GA, LB, RP, and FS conceived and designed the experiments. NM, EM, RP, and FS performed the experiments. NM, JN, and FS analyzed the data. NM, GM, EA, FR, GA, LB, EM, JN, RP, and FS wrote the manuscript. All authors read and approved the final version of the manuscript.

## Conflict of Interest

The authors declare that the research was conducted in the absence of any commercial or financial relationships that could be construed as a potential conflict of interest.

## Footnotes

1 http://www.ncbi.nlm.nih.gov/

2 www.clcbio.com

3 http://imed.med.ucm.es/Tools/sias.html

4 https://www.ncbi.nlm.nih.gov/orffinder/
