# Supplementary material for: Metagenomic Analysis of Plant Viruses Associated With Papaya Ringspot Disease in Carica papaya L. in Kenya
Source: Front Microbiol. 2020 Mar 4;11:205. doi: 10.3389/fmicb.2020.00205 (PMC7064807; doi:10.3389/fmicb.2020.00205)
Supplement: Supplementary file 1 [file Image_1.pdf]

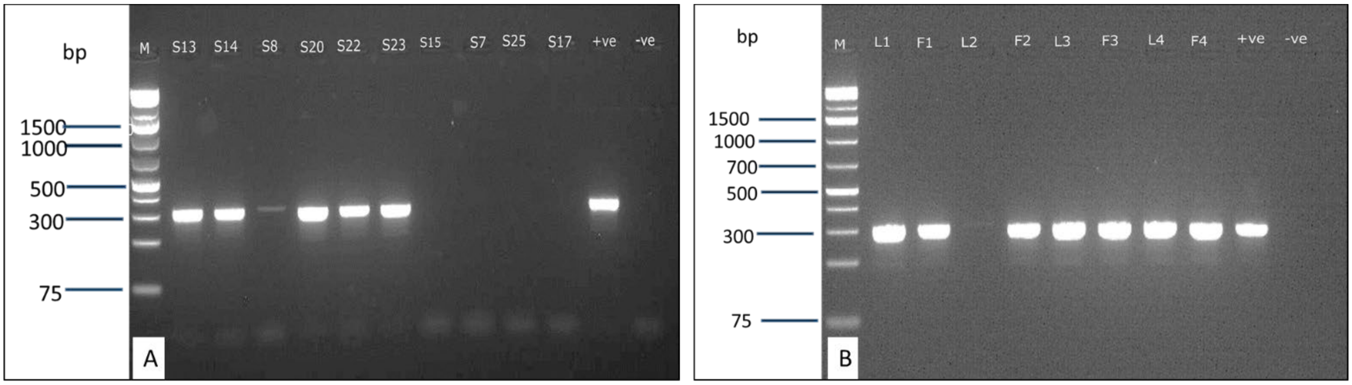

Supplementary Figure S1: Examples of the use of RT-PCR to confirm the presence of MWMV detected by Illumina sequencing in sampled papaya leaf tissues and in ringspots extracted from fruits. A band at 315bp in A and B represented presence of MWMV M= O'GeneRuler ™ 1-Kb plus DNA ladder, +ve = positive control, -ve=negative control. Numbers S13-S17=papaya samples labelled according to their sequencing number (Table 1). F1, F2, F3 and F4 represents virus extracted from the ringed spots on fruits while L1, L2, L3 and L4, represent virus extracted from leaves.
