# Supplementary material for: Metagenomic Analysis of Plant Viruses Associated With Papaya Ringspot Disease in Carica papaya L. in Kenya
Source: Front Microbiol. 2020 Mar 4;11:205. doi: 10.3389/fmicb.2020.00205 (PMC7064807; doi:10.3389/fmicb.2020.00205)
Supplement: Supplementary file 2 [file Table_1.DOCX]

**Supplementary Table S1**: Illumina MiSeq sequencing statistics obtained from papaya samples with and without PRSD symptoms from 22 counties in Kenya

| **Sample No** | **County of collection** | **Raw reads** | **Length (bp) before QC** | **Trimmed reads** | **Length (bp) after QC** | **% GC content** | **Virus*** |
| --- | --- | --- | --- | --- | --- | --- | --- |
| S1 | Makueni | 465116 | 35-151 | 436423 | 60-151 | 44 | + |
| S2 | Makueni | 878230 | 35-151 | 824664 | 60-151 | 46 | + |
| S3 | Kiambu | 484883 | 35-151 | 450236 | 60-151 | 45 | – |
| S4 | Nakuru | 992553 | 35-151 | 927987 | 60-151 | 46 | + |
| S5 | Nakuru | 840783 | 35-151 | 788212 | 60-151 | 45 | + |
| S6 | Baringo | 1045411 | 35-151 | 981992 | 60-151 | 45 | + |
| S7 | Taita Taveta | 678903 | 35-151 | 638032 | 60-151 | 44 | – |
| S8 | Homabay | 608410 | 35-151 | 569076 | 60-151 | 44 | + |
| S9 | Kwale | 818104 | 35-151 | 770565 | 60-151 | 44 | – |
| S10 | TaitaTaveta | 882906 | 35-151 | 832445 | 60-151 | 42 | + |
| S11 | Kiambu | 1145487 | 35-151 | 1073453 | 60-151 | 45 | + |
| S12 | Kitui | 1107196 | 35-151 | 1042103 | 60-151 | 42 | + |
| S13 | Busia | 781327 | 35-151 | 733690 | 60-151 | 44 | + |
| S14 | Kisumu | 1240288 | 35-151 | 1167504 | 60-151 | 44 | + |
| S15 | Taita Taveta | 587524 | 35-151 | 551297 | 60-151 | 44 | + |
| S16 | Kilifi | 895032 | 35-151 | 842086 | 60-151 | 46 | + |
| S17 | Makueni | 1080677 | 35-151 | 1018184 | 60-151 | 44 | + |
| S18 | Kitui | 1075787 | 35-151 | 1004375 | 60-151 | 44 | – |
| S19 | Murang’a | 1288194 | 35-151 | 1212378 | 60-151 | 45 | + |
| S20 | Murang’a | 845593 | 35-151 | 791310 | 60-151 | 46 | + |
| S21 | Kirinyaga | 1082909 | 35-151 | 1016693 | 60-151 | 45 | + |
| S22 | Kirinyaga | 1118095 | 35-151 | 1053334 | 60-151 | 44 | + |
| S23 | Embu | 1309867 | 35-151 | 1237337 | 60-151 | 42 | + |
| S24 | Embu | 831762 | 35-151 | 782762 | 60-151 | 44 | + |
| S25 | Kwale | 1207561 | 35-151 | 1153706 | 60-151 | 44 | + |
| S26 | Machakos | 933695 | 35-151 | 900984 | 60-151 | 45 | + |
| S27 | Homabay | 1124740 | 35-151 | 1080401 | 60-151 | 46 | + |
| S28 | Embu | 1236829 | 35-151 | 1183709 | 60-151 | 45 | – |
| S29 | Tharaka Nithi | 1289557 | 35-151 | 1243865 | 60-151 | 42 | + |
| S30 | Tharaka Nithi | 600317 | 35-151 | 578568 | 60-151 | 48 | – |
| S31 | Meru | 1110859 | 35-151 | 1068658 | 60-151 | 45 | + |
| S32 | Meru | 729236 | 35-151 | 702649 | 60-151 | 45 | – |
| S33 | Meru | 1387548 | 35-151 | 1329158 | 60-151 | 44 | + |
| S34 | Meru | 1373080 | 35-151 | 1320794 | 60-151 | 44 | + |
| S35 | Kiambu | 698466 | 35-151 | 668678 | 60-151 | 45 | + |
| S36 | Kiambu | 1060572 | 35-151 | 1021341 | 60-151 | 45 | + |
| S37 | Kilifi | 1142123 | 35-151 | 1098926 | 60-151 | 46 | – |
| S38 | Baringo | 1635383 | 35-151 | 1560252 | 60-151 | 45 | – |
| S39 | Kitui | 1401904 | 35-151 | 1352125 | 60-151 | 44 | + |
| S40 | Kisumu | 955874 | 35-151 | 918727 | 60-151 | 45 | + |
| S41 | Makueni | 802399 | 35-151 | 766667 | 60-151 | 46 | + |
| S42 | Kirinyaga | 1174298 | 35-151 | 1132021 | 60-151 | 44 | + |
| S43 | Machakos | 1809690 | 35-151 | 1746884 | 60-151 | 43 | + |
| S44 | Machakos | 1151750 | 35-151 | 1089401 | 60-151 | 45 | + |
| S45 | Makueni | 1727221 | 35-151 | 1656070 | 60-151 | 44 | + |
| S46 | Murang’a | 1413519 | 35-151 | 1362841 | 60-151 | 44 | + |
| S47 | Kirinyaga | 1395345 | 35-151 | 1347231 | 60-151 | 45 | + |
| S48 | Elgeyo Marakwet | 800266 | 35-151 | 770949 | 60-151 | 45 | – |
|  |  | **50,247,269** |  | **47,800,743** |  |  |  |

Key: T. Taveta – Taita Taveta, T. Nithi – Tharaka Nithi, E. Marakwet – Elgeyo Marakwet. *Presence or absence of virus is indicated by + or – respectively
